# Beyond Covalent Crosslinks: Applications of Supramolecular Gels

**DOI:** 10.3390/gels4020040

**Published:** 2018-05-03

**Authors:** Ty Christoff-Tempesta, Andrew J. Lew, Julia H. Ortony

**Affiliations:** 1 Department of Materials Science and Engineering, Massachusetts Institute of Technology, Cambridge, MA 02139, USA; ty.christoff@mit.edu; 2 Department of Chemistry, Massachusetts Institute of Technology, Cambridge, MA 02139, USA; lewan@mit.edu

**Keywords:** supramolecular gel, self-assembly, gels, applied soft matter

## Abstract

Traditionally, gels have been defined by their covalently cross-linked polymer networks. Supramolecular gels challenge this framework by relying on non-covalent interactions for self-organization into hierarchical structures. This class of materials offers a variety of novel and exciting potential applications. This review draws together recent advances in supramolecular gels with an emphasis on their proposed uses as optoelectronic, energy, biomedical, and biological materials. Additional special topics reviewed include environmental remediation, participation in synthesis procedures, and other industrial uses. The examples presented here demonstrate unique benefits of supramolecular gels, including tunability, processability, and self-healing capability, enabling a new approach to solve engineering challenges.

## 1. Introduction

Exciting material architectures with unconventional blends of properties exist between the domains of traditional solids, liquids, and gases. In particular, gels are a dispersion of fluids within a solid, which are able to retain a viscoelastic solid-like form despite a majority portion of their mass usually being comprised of some liquid [1]. Paul Flory established four canonical categories of gels in 1974 [2]: (1) high-order lamellar gels (e.g., phospholipids), (2) disordered covalent networks (e.g., vulcanized rubber), (3) semi-ordered physical networks (e.g., gelatin), and (4) disordered particulate gels (e.g., reticular fiber networks) [1]. Gels can be further distinguished by the type of solvent used to yield categories such as hydrogels, organogels, and aerogels.

Supramolecular gels are assembled from distinct constituent molecules, and have been a focus of increased research since the turn of the century [3]. In the strictest sense, they are a subcategory of Flory gel category 4, and are comprised of low molecular weight gelators [4] self-assembled into a disordered network structure through secondary noncovalent bonds, such as solvophobic interactions, π–π stacking, and hydrogen bonding, among others. The distinction of secondary bonding among molecular units differentiates supramolecular gels and traditional covalently bonded network gels, creating unique properties and functionalities that utilize its ability to more easily and reversibly break and reform bonds between subunits.

**Figure 1:**
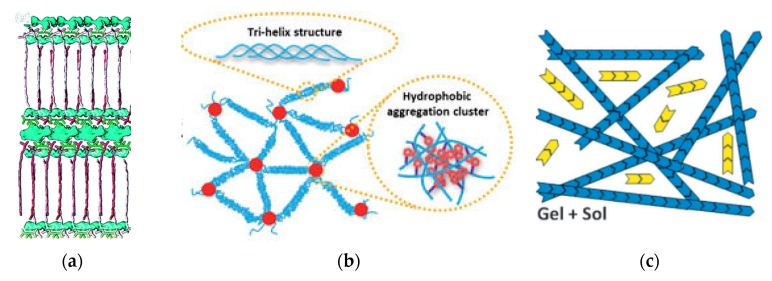
Examples of supramolecular gels are found in three of the four categories of gels defined by Flory [2]: (**a**) category 1: high-order lamellar gels [6]. Adapted with permission from ref [6]. Copyright 2016 Boekhoven et al.; (**b**) category 3: semi-ordered physical networks [7]. Adapted with permission from ref [7]. Copyright 2018 Springer Nature; and (**c**) category 4: disordered particulate gels [5]. Adapted with permission from ref [5]. Copyright 2012 Royal Society of Chemistry; The second category identified by Flory, disordered covalent networks, is not considered to be supramolecular.

However, the moniker of supramolecular gels has also been attributed less strictly by many authors to mean any gel utilizing noncovalent bonding between molecular species [5]. Thus, supramolecular gels can be taken to include even polymeric gels with covalent bonding so long as they include other species that are noncovalently bonded to the gel, ionic crosslinks, or physical bonding between polymeric chains (as opposed to exclusively covalent crosslinks). In this view, certain gels within Flory gel categories 1 and 3 as well as assemblies like gel nanocomposites have also been categorized as supramolecular. For the purposes of reporting the wide variety of possibilities in this developing field, here we take a wide approach to supramolecularity and include all of the above into consideration (Figure 1).

Supramolecular gels generally have more parameters that can be adjusted to finely tune their properties than their non-supramolecular counterparts. Differences in the eventual mechanical, electrical, and biological properties of the resultant gel are derived from not only the conventional considerations of solvent identity and gelator concentrations, but the utilization of differently functionalized molecular units, the incorporation of additives into the supramolecular matrix, and modifications into self-assembly behavior.

While the boundaries of this material class have previously been vaguely defined, the promise of novel structure-property interactions has spurred exploration into the extent of their use. In this review, we first discuss how supramolecular gels can improve upon traditional polymeric conductors, allow tunable access to unique light-matter interactions, and open new paradigms of optoelectronic devices. The impact of supramolecular gels on advancements in alternative energy is explored next from both the energy generation and energy storage angles. Then, improvements over existing biomedical technologies are discussed through the lens of novel supramolecular gel capabilities in drug delivery, tissue engineering, and paradigms for wound treatment and non-viral vector gene delivery. The capacity to reshape key biological techniques is subsequently discussed, including cell storage and study, electrophoresis, and indication of biological compounds. A further collection of emerging applications is also treated (e.g., environmental remediation and surface coatings, among others), as we look toward the future of this developing field.

Within each section, selected gelators from the presented literature are provided to highlight the diversity of structures used in forming supramolecular gels; these structures are denoted throughout the text. For further information about the synthesis or processing of a specific gelator, readers are directed to the original article.

## 2. Supramolecular Gels for Optoelectronic Applications

### 2.1. Electrical Conduction

In the realm of electronics, efforts have been raised to utilize the unique properties of supramolecular gels for next-generation electrical conductors. Traditional conductive polymers suffer from reduced flexibility [8] and processability [9] compared to non-conductive polymers, limiting their potential use in desired applications. The use of supramolecular gels for electronic applications has been demonstrated as a pathway toward alleviating these problems and adding extra functionality. Here we touch on three beneficial aspects of electrically conductive supramolecular gels: their flexibility, processability, and self-healing properties.

#### 2.1.1. Flexibility

Conductive gels in chloroform solvent were synthesized by Pépin-Donat et al. in a reported series of conjugated covalent poly-octylthiophene (POT) gels. Formed by the copolymerization of octylthiophene with trithienylbenzenes, followed by swelling with chloroform and doping with iodine, the resulting gels acted as electrically conductive rubbers due to the external ionic dopant [8]. While these still possessed polymeric crosslinks, other approaches have led to flexible supramolecular conductors like the category of nanocomposite bucky gels in imidazolium-ion-based ionic liquids, which are formed by the interactions between imidazolium ions and π-electronic surfaces of ground carbon nanotubes [10,11]. Ionic liquid-based triblock copolymer gels, for example in butylmethylimidazolium phosphorus hexafluoride as in the work of Lodge et al., have also been identified as flexible conductors by Kang et al. The introduction of ABA triblock copolymers in an ionic liquid with miscible central B blocks and immiscible end A blocks results in a self-assembled gel network of connected micellar structures [12]. These flexible and conductive supramolecular gel structures are suitable for applications such as electrical actuators for artificial muscles [13,14], electromechanical sensors [15], and electrolytes for flexible supercapacitors [16].

#### 2.1.2. Processability

Gel processing techniques such as the one-pot melt-blending gelation used by Kim et al. provide great advantages in the scalability and cost of electrically conducting materials. The utilization of a eutectic liquid of diphenylamine and benzophenone in the synthesis of bucky gels allows for the dissolution of polymers and gelation of carbon nanotubes, bypassing long and often toxic alternative processes [17]. Other supramolecular gels, like the aforementioned triblock copolymer gels, have the added benefit of reversible formation, with temperature [12,18] or light (demonstrated with UV [12] or X-ray [19]) acting as reversible and controllable triggers for gelation. This capability allows for easier downstream processing (e.g., alterations for specific applications) with these gels than with traditional conductive polymers like polypyrrole [9] or polyaniline [20], which are difficult to further process due to their mechanical rigidity and low solubility. Processing by three-dimensional (3D) printing has been achieved with poly(*N*-acryloyl glycinamide) [21] and H2010h1 [22] hydrogels, adding further versatility to a supramolecular gel approach.

#### 2.1.3. Self-Healing

**Figure 2:**
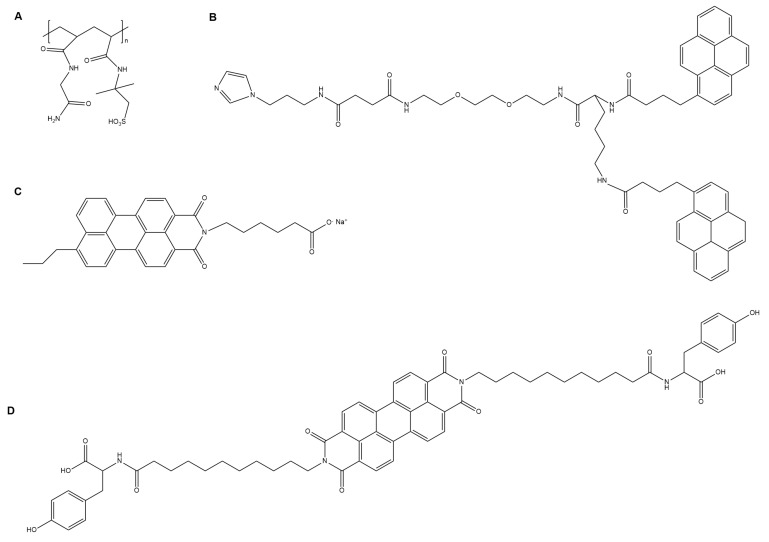
Selected gelators developed for optoelectronic applications: (**A**) a conductive, self-healing PNAGA-PAMPS/PEDOT/PSS-3-49 gelator [21]; (**B**) a fluorescent gelator with hydrophobic moieties of pyrenebutyric acid and a hydrophilic moiety of an ethyleneoxy unit coupled with 1-(3-aminopropyl)imidazole [23]; (**C**) a photocatalytic chromophore amphiphile gelator [24]; and (**D**) a dipeptide-based perylene bisimide derivative gelator whose xerogel exhibits current photo-switching properties [25].

**Figure 3:**
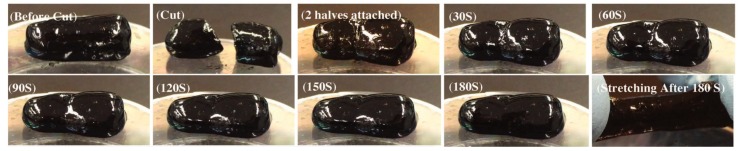
The conductive H2010h1 hydrogel by Darabi et al. exhibits self-healing over minutes (seconds since cutting shown in inset) with, importantly, the restoration of mechanical and electrical properties [22]. Adapted with permission from ref [22]. Copyright 2017 John Wiley and Sons.

The capacity for self-healing behavior, in conjunction with flexibility, conductivity, and processability, further expands the scope of applications of conductive supramolecular gels. Work by Wu et al. quantified the healing response in a PNAGA-PAMPS/PEDOT/PSS-3-49 hydrogel (structure shown in Figure 2), noting that immersion in a 90 °C water bath for several hours mended a sample of gel that had previously been cut in half and restored both flexible physical and conductive electrical properties. Hydrogen bond-based crosslinking allowed for healing after both multiple cuts and prolonged times between cuts [21]. Darabi et al. described self-healing in the aforementioned H2010h1 hydrogel two minutes after cut pieces were brought into contact (Figure 3). This was hypothesized to occur because of ionic interactions (between ferric ions and carboxylic groups of poly(acrylic acid)) and hydrogen bonding (between chitosan and poly(acrylic acid)) within the hydrogel [22].

Such materials can be used not only for flexible electrodes or sensors as previously stated, but also for self-repairing circuits and electroactive scaffolding for soft tissue engineering [21]. Artificial skin and biomimetic prostheses are a favored biological application where the conduction of electronic signals is critical for functioning [22,26]. Further research efforts are directed towards balancing the benefits of the aforementioned soft, self-healing type properties with even more robust mechanical properties for applications in fields like soft robotics [27] and smart wearables [28]. Biomedical applications in particular form an exciting frontier for supramolecular gels, and will be further detailed in a later section.

### 2.2. Photonic Responses

Beyond electrical conduction, supramolecular gels have been materials of interest in photonic applications because of their ability to control their interactions with light. Light-matter interactions are of primary importance in informational, optical, and photocatalytic applications, among others. The deliberate engineering of specific categories of light responses provides a suite of materials able to perform a wide breadth of functions usually associated with other material classes. Specific engineered light-matter interactions covered here include the luminescence, scattering, refraction, and absorbance of light.

#### 2.2.1. Luminescence

A variety of gels have been shown to emit different spectra of light via various modes. For instance, in investigations of low molecular weight supramolecular gelators, Mandal et al. formed a fluorescent gelator with hydrophobic moieties of pyrenebutyric acid and a hydrophilic moiety of an ethyleneoxy unit coupled with 1-(3-aminopropyl)imidazole (structure shown in Figure 2). The mixture of these compounds formed both hydrogels and nitrobenzene organogels and, due to the pyrene moieties, emitted blue light from its fiber network under ultraviolet (UV) radiation [23]. Strong blue fluorescence upon UV exposure has also been observed from an acylhydrazone-functionalized dual benzimidazole derivative supramolecular organogel with dimethyl sulfoxide (DMSO)-ethylene glycol (1:3) or dimethylformamide (DMF)-water (1:5) solvent, enhanced with one equivalent of rare earth Y^3+^ [29].

More general luminescence has been observed with a similar acylhydrazone-functionalized benzimidazole derivative assembled in ethylene glycol and (for a Job plot experiment) DMF-water. When cooled to room temperature, this gel luminesced green, which could be tuned to blue with the addition of one equivalent of Cd^2+^ [30]. Luminescence has also been observed with 8-hydroxyquinoline lithium (LiQ) in non-protic solvents like benzene or toluene, with a 3-fold increase in luminescence when modified with cholesterol and a red shift in absorption when gelled into a J-aggregate [31]. Kishimura et al. demonstrated red luminescence in an organogel formed from a trinuclear Au(I) pyrazolate complex in hexane, which could be tuned to blue upon doping with Ag^+^, and green upon subsequent heating [32].

Such materials have been explored for sensors, due to the fact that the wavelengths of light emitted can change in the presence of various ions. Lin et al. reported one such system, most stable in ethanol, that fluoresces when the gel is coordinated with Pb^2+^ ions. Upon testing 14 anions, it was discovered the system’s fluorescence could be selectively quenched only with I^−^. Further addition of Pb^2+^ to the system allowed for the effect to be reversed [33]. Two further supramolecular gels investigated by Lin et al., which operate in a similar fashion and are also most stable in ethanol, when coordinated (1) with Mo^2+^ and Co^2+^ and (2) with Ca^2+^ and Fe^3+^ could reversibly and visually sense Cl^-^ and H_2_PO_4_^−^, respectively [34,35]. In a complementary line of work, Cu^2+^ was detected by a green to blue color transition in a supramolecular thiazolothiazole derivative gel in cyclohexanone by Zhang et al. [36] or a yellow color to colorless transition in a salen-linked gelator in DMSO-water (1:1) by Fan et al. [37]. Catalysis monitoring has been explored in the work of Harris et al. with a sensor-functionalized gelator that gels in various solvents like DMF, i-PrOH, and water, increasing the local fluorescence in the presence of a desired reaction product [38].

The range of possible emission colors has piqued interest in applications toward information storage and display. One frequently cited application is for rewritable, inkless security paper, achievable through the toggling of fluorescence properties via triggers like mechanical grinding, exposure to solvent vapors, electric fields [39], heat [40], and UV light [41].

#### 2.2.2. Scattering

Beyond the emission of light, supramolecular gels have been synthesized to scatter light in deliberate and useful ways. Structural color was achieved by Zhou et al. with supramolecular organogels co-assembled with oleophilic silica nanoparticles and carbon black in a hot chloroform-silicone oil solution. This usage of a self-healing gel architecture for a structural color material resulted in the first reported non-iridescent photonic material that can restore both its modulus and color properties after mechanical damage, i.e., cuts and shear failure. Proposed applications include not only the types of chemical sensing mentioned previously, but also flexible color displays [42].

Light scattering supramolecular gels have also been formed by polyfluorene-based π-conjugated polymers. Chen et al. synthesized liquid crystal physical gels using poly(9,9-dioctylfluorene-alt-benzothiadiazole) (F8BT) gelators in nematic liquid crystal E7 (from Fusol material) via a cooling process from isotropic melt. Such a structure possesses the ability for anisotropic scattering behavior due to the fibrillar aggregation of F8BT, with more scattering occurring for light polarized parallel to the fibers. Notably, this structure is able to switch modes from being transparent to exhibiting anisotropic scattering via the application of a low (2.7 V) driving voltage—much easier than for other liquid crystal gels, which require around 20–60 V [43,44]. Upon the removal of the driving field, the gel returns to being transparent. Such a triggerable, reversible scattering response has potential applications as a smart polarizer or in transflective display technology [45].

#### 2.2.3. Refraction and Absorption

**Figure 4:**
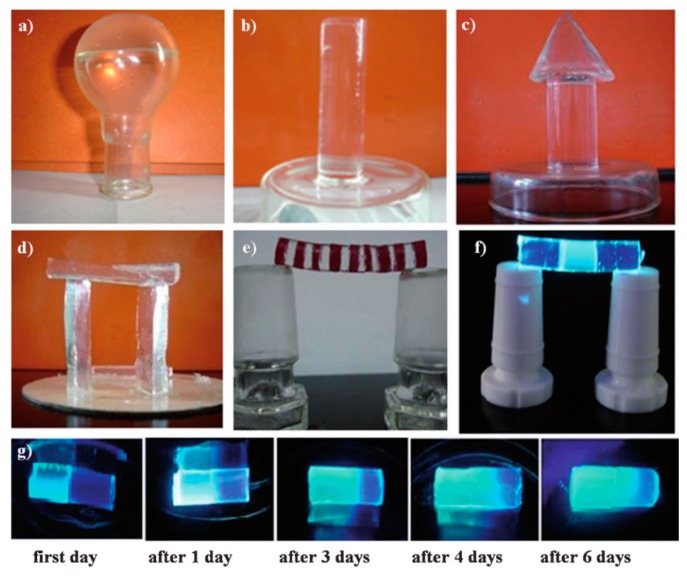
Vidyasagar et al. organogels combine glass-like optical properties with desirable physical properties, including moldability (shown by various shapes in (**a**–**d**)), robustness (shown in (**e**,**f**)), and self-healing (shown by UV doping one tube of a self-assembling gel before attaching to another in (**g**)) [46]. Reprinted with permission from ref [46]. Copyright 2011 John Wiley and Sons.

Other self-healing supramolecular gels have been developed to transmit and refract light. Using mannitol-based diols flanked by ketal moieties, Vidyasagar et al. produced transparent supramolecular organogels with a variety of alkane and aromatic organic solvents (Figure 4). These gels possess the favorable physical properties of flexibility, moldability, and self-healing combined with optical properties of visible light transmittance, UV light filtration, and glass-like indices of refraction. This wide variety of useful properties has allowed for demonstrated applications in self-healing, shatter-proof, and scratch-free lenses and prisms in place of glass, as well for proposed applications in UV-protective goggles or potential replacements of the oils used in various measurement techniques such as oil immersion microscopy and differential interference contrast microscopy [46].

There also has been research in the direction of gels that absorb light. Following up on the 2014 work by Weingarten et al. [47], supramolecular chromophore amphiphile hydrogels (structure shown in Figure 2) in the 2017 work of Kazantsev et al. demonstrated a photocatalytic effect. The crystalline phase of this material allows for the formation of excitons under visible light, aiding hydrogen production by the increased photosensitization of a proton reduction catalyst such as [Mo_3_S_13_]^2−^ [48]. Another supramolecular gel shown to absorb light was formed from disubstituted acenes. These organogels were shown to self-assemble with a 2,3-di-n-decyloxy derivative gelator into fibrillar structures in a wide range of organic solvents, aligned by magnetic fields or mechanical force, and act as a light-harvesting matrix and optical confining media [24]. As might be expected with such a host of electronic and optically relevant properties, supramolecular gels have the potential to impact a variety of optoelectronic fields, with more work to be done in gel stability and device manufacture.

### 2.3. Optoelectronic Devices

#### 2.3.1. Semiconductors

A critical foundation of modern electronic devices is the semiconductor. Traditionally inorganic materials (historically germanium and silicon [49], with more recent advancements in III-V compounds like GaAs and InN [50]) have been used in the semiconductor industry, but organic semiconductors have been on the rise in the search for flexible [51], cheaper [52], and potentially more environmentally friendly materials. In particular, semiconductive supramolecular gels have been explored over the past decade.

**Figure 5:**
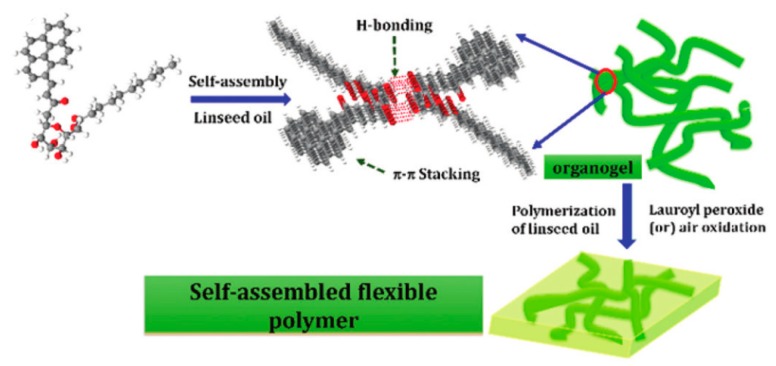
Hierarchical assembly of a semiconductive organogel by Muthusamy et al. from bio-based plant oils, which could be formed into flexible sheets for use as a charge transport layer in organic field effect transistors [54]. Adapted with permission from ref [54]. Copyright 2016 Royal Society of Chemistry.

As an example, Muthusamy et al. produced semiconductive fluorescent organogels from the direct condensation of vinyl esters with functionalized sugar alcohol, using hydrophobic solvents and the lipase Novozyme 435. Doping with graphene resulted in an increase in the current response for a given applied voltage, up to a 1:1 ratio of gel to graphene, allowing the system’s semiconductive behavior to be tuned. Their particular process used bio-based plant oils, which have been identified as a particularly economical and renewable category of industrial chemicals [53]. Flexible sheets of this material were made by the auto-oxidation of linseed oil, making it useful as a charge transport layer in organic field effect transistors (Figure 5) [54]. Earlier work by Diring et al. utilizing hydrogen bonding from amide groups to form luminescent ethynyl-pyrene gels in DMF, toluene, and cyclohexane resulted in a field effect transistor prototype with favorable source-to-drain electron/hole transport. While applied gate voltage only negligibly affected the source-to-drain current, making for an insufficient transistor [55], the properties of the formed gel were still favorable for other applications, such as fiber-based organic light emitting diodes [56]. Further work in this field is directed toward increasing the performance of these devices to match those of traditional semiconductors.

#### 2.3.2. Logic Gates and Photo-switching

A computation-focused application of semiconducting supramolecular gels is in the realm of gel-based logic gates. The work of Roy et al. in 2014 demonstrated a dipeptide-based perylene bisimide derivative (PBI-C_11_-Y) (structure shown in Figure 2) that self-assembled into a hydrogel between pH 7.46 to pH 9 and exhibited both semiconductivity and, notably, current photo-switching behavior in its xerogel state. Like the previous semiconducting gel example from Muthusamy et al., the optoelectronic response of this gel can be tuned with the inclusion of graphene. Specifically, incorporation of graphene oxides into the gel allows for a more distinct “on” current state to be accessed upon white light exposure, opening the possibility of a logic gate-esque system based on these self-assembled gels [25]. However, such a material was only shown to cycle four times before a roughly 33% degradation in distinction between “on” and “off” states. Further instances of potential logic gate behavior via photo-switching occurred, for example, with a low molecular weight organogelator mixed with trioctylphosphine oxide ligand-capped CdSe/ZnS core-shell nanoparticles in *n*-hexane. This luminescent, transparent organogel was synthesized in 2016 by Schmidt et al. and, with the incorporation of diarylethene, exhibited UV-vis light triggered photo-switching. Notably, diarylethene’s intrinsic photochromic properties were retained within the organogel structure with over 20 cycles of reversible switching demonstrated [57], making it more robust than the previous example.

While the prior examples have exhibited triggered photo-switching behavior, they alone have not exhibited the ability to simulate specific logic gates such as AND or OR. That said, work by Jung et al. showed a Co^2+^-imidazole-based supramolecular gel in polar solvents such as DMF, DMF-water (1:1), and DMA with multifactor dependent photo-switching effects. Specifically, the gel possesses an ionochromic effect, turning from red to blue with the addition of Cl^−^ or Br^−^ ions, and a piezochromic effect, turning from red to blue when ground with KBr powder [58]. The ionochromic effect acts as an OR gate because it requires either or both of the ions’ presence to change colors, while the piezochromic effect emulates an AND gate as it demands both the presence of KBr powder and pressure to change color. However, the necessity of mechanically grinding the gel with KBr powder for the piezochromic effect appears to be materially costly and impractical for any large calculation, compared to using conventional transistor-based devices. Thus, while supramolecular gel systems have been explored in proof-of-concept designs for novel optoelectronic switches, progress appears to still be in early stages of development.

Another large class of optoelectronic device applications for semiconducting supramolecular gels, hinted at with the variety of gels previously shown to absorb light, is the photovoltaic cell. Supramolecular gel properties of tunable electrical conductivity and photon absorbance synergize to form an enticing material platform for solar capture and conversion to current. Further perspectives on photovoltaic cells will be detailed in the following section dedicated to energy applications.

## 3. Supramolecular Gels for Energy Applications

### 3.1. Energy Generation

A large section of supramolecular gel applications is devoted to the energy sector. Within this realm, solar cells are a particularly enticing application due to the tunability of supramolecular gels’ electrical and optical properties. As an example, in the work of Bairi et al., a xerogel was formed by adding anilinium chloride solution and ammonium persulfate solid to a 5,5′-(1,3,5,7-Tetraoxopyrrolo[3,4-f]isoindole-2,6-diyl)-diisophthalic acid (PMDIG) hydrogel. Due to the *p*-type nature of polyaniline (PANI) and the *n*-type PMDIG, the resultant PMDIG-PANI xerogel possessed a *p*-*n* junction with an I–V curve that showed photocurrent rectification under exposure to white light. When incorporated into an ITO/PMDIG-PANI/graphite device, a power conversion efficiency of 0.1% was obtained [59], demonstrating proof of concept but also signifying the gap between gel and traditional solar cell performances.

**Figure 6:**
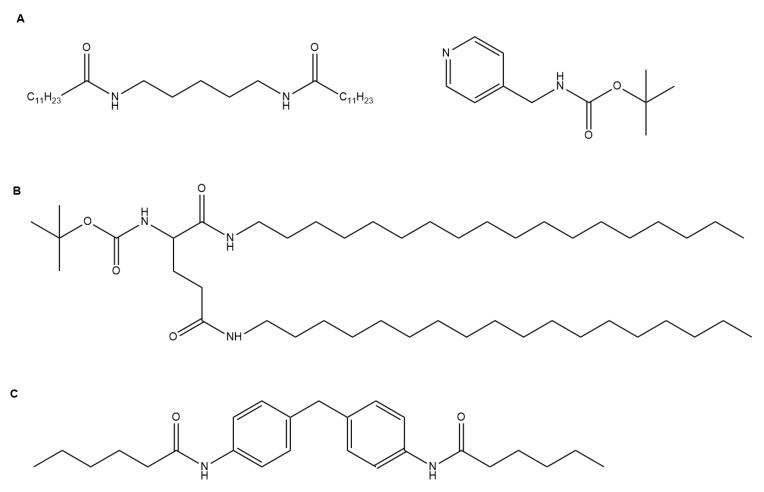
Selected gelators developed for energy applications: (**A**) Co-assembled *N*′-1, 5-pentanediylbis-dodecanamide and 4-(Boc-aminomethyl)pyridine gelators for solar energy generation [61]; (**B**) a thylakoid membrane-inspired organogel for photon up-conversion [63]; and (**C**) a supercapacitor bis(4-acylaminophenyl)methane gelator [64].

Supramolecular gels have also found uses in advancing dye-sensitized solar cell technology, serving as an enticing replacement material for liquid electrolytes due to their conductivity and flexibility without the risks of liquid leakage [60]. In 2015, Huo et al. demonstrated these benefits with their two-component quasi-solid-state electrolyte gel made from *N*,*N*′-1, 5-pentanediylbis-dodecanamide and 4-(Boc-aminomethyl)pyridine gelators (structures shown in Figure 6) in liquid electrolyte 0.1 M iodine (I_2_), 0.1 M anhydrous lithium iodide (LiI), 0.5 M *N*-methylbenzimidazole (NMBI), and 1 M DMPII in 3-methoxypropionitrile (MePN). This two-component electrolyte resulted in a solar cell with a power conversion efficiency of 7.04%, comparable to the baseline of 7.11% for liquid electrolytes and greater than the baseline of 6.59% for a single-component *N*,*N*ʹ-1, 5-pentanediylbis-dodecanamide electrolyte [61]. Huo et al. further reinforced the field in 2017, this time with a slightly different suite of materials: *N*,*N*′-1,8-octanediylbis-dodecanamide and iodoacetamide in a liquid electrolyte 0.1 M LiI, 0.6 M I_2_, 0.45 M NMBI, and 0.9 M MePN. Again comparing a two-component versus a single-component system, the two-component electrolyte gave a conversion efficiency of 7.32% versus the single-component electrolyte’s 6.24% [62]. Such results are comparable with the previous set of materials, but illustrate a power conversion efficiency surpassing that of the baseline liquid electrolyte.

**Figure 7:**
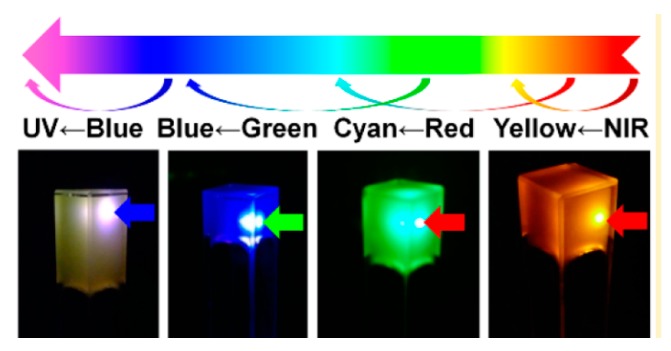
A series of organogels from Duan et al., who performed photon up-conversion for wavelengths across the visual spectrum (color of incident wavelength shown in arrow) [63].

Tackling the energy issue from another angle, supramolecular gels have also been used for photon up-conversion. Up-conversion has the potential to increase the efficiency of solar cells by allowing photons with energies less than the semiconductor band gap to contribute to exciton generation [65,66,67]. Duan et al. used supramolecular organogels in DMF to perform triplet-triplet annihilation (TTA)-based up-conversion (UC) (Figure 7). The usual problems with TTA-UC of triplet deactivation via oxygen exposure or the necessity for high power illumination [68] were sidestepped with the supramolecular gel architecture, which shielded donor/acceptor molecules from oxygen deactivation while also presenting efficiently transferred triplet energy. Previous to this work, conventional ways of ameliorating oxygen deactivation would often exacerbate the need for high-power light [63]. This utilization of a supramolecular organogel, inspired by thylakoid membranes in natural light harvesting systems (structure shown in Figure 6), allowed for the formation of a photon up-conversion system incorporating benefits once thought to be exclusive of each other. Equally important to the generation of energy is the issue of energy storage, which will be discussed in detail in the following section.

### 3.2. Energy Storage

Different technologies of energy storage span a range of specific powers and specific energies, suited to a variety of different applications. The three main energy storage applications detailed here that supramolecular gels have found significant uses in are Li-ion batteries, supercapacitors, and fuel cells.

#### 3.2.1. Li-Ion Batteries

Supramolecular gels can impact the battery industry, expanding the range of effective battery applications. Work by Frischmann et al. detailed a system that uses a perylene bisimide-polysulfide (PBI-PS) gel from catholyte solutions containing 2.5 M S as Li_2_S_8_ and 0.048 M PBI (5.0% *w*/*w*) in tetraethylene glycol dimethyl ether (TEGDME) with 0.50 M lithium bis(trifluoromethanesulfonyl)imide (LiTFSI) and 0.15 M LiNO_3_ to improve upon lithium-sulfur hybrid redox flow batteries. π-conjugated organic redox mediators, inspired by nanocarbon current collectors in other systems [69], were investigated to assist with charge-transport bottlenecks. Computational simulation identified the aforementioned PBI-PS gel as favorable because it exhibited a similar charge/discharge potential to Li-S as well as the ability to form durable nanowire structures. As a self-assembled, nanostructured, and flowable gel, PBI-PS has been shown to improve sulfur utilization and have potential for opening usage to both longer and larger-scale battery applications [70].

Supramolecular gels also have potential in advancing battery electrode technology. Metal oxide nanoparticles can be enticing electrodes for Li-ion batteries [71], but periodic volume changes and structural stress due to repeated Li insertion can lead to nanoparticle aggregation and limits on their functional lifetime. Synthetic procedures for hollow sphere structures in supramolecular gels have been investigated for their ability to accommodate space for Li and mitigate these expansive stresses [72]. In the work of Lyu et al., a supramolecular hydrogel was used as a template for nanoparticle anode fabrication. Hollow CuO nanoparticles were formed via directed self-assembly in a mixture of chitosan, ascorbic acid, and Cu^2+^ ions, with subsequent calcining at 500 °C. The resultant composite exhibited over 90% retention of discharge capacity after 500 cycles, considered an excellent cycling performance [73]. Additionally, Sui et al. reported using supramolecular gels as guiding matrices for manganese dioxide deposition. This resulted in MnO_2_/nitrogen-doped graphene hybrid aerogels with increased Li cycling resistance that could similarly be used as an anode material for a Li-ion battery [74].

Another method to combat the problem of device degradation after repeated Li-insertion-driven volume changes has been to prevent nanoparticle aggregation directly. Shi et al. synthesized a supramolecular hydrogel binder as a coating for anodic SnO_2_ nanoparticles. This gel was formed via reversible ionic bonding between poly(allylamine hydrochloride) (PAH) chains and a phytic acid gelator, and thus exhibited self-healing properties. As an added benefit, the addition of carbon nanotubes into the gel was shown to improve the specific capacity of the battery. The presence of this supramolecular binder coating acted to suppress the aggregation of SnO_2_ particles, and its ability to self-heal allowed for a robust structure capable of withstanding volume changes after repeated Li insertion cycles. Specifically, the specific capacity of an uncoated SnO_2_ sample was 169 mAh/g after 70 cycles, compared to a coated 60 wt % PAH sample, which retained a specific capacity around 500 mAh/g after 70 cycles [75].

Though there have been a variety of studies primarily on Li-ion battery technology, as further work is conducted in this field other types of batteries have the potential to also benefit from supramolecular gels.

#### 3.2.2. Supercapacitors

Aside from traditional batteries, supercapacitors have been a popular device for supramolecular gel research. This is due to supercapacitors’ ability to bridge the gap in properties between the high specific power but low specific energy of conventional capacitors and the low specific power but high specific energy of batteries [76]. As briefly mentioned previously with the work of Kang et al., supramolecular gels have been studied as electrolytes for flexible supercapacitors. Focused work into the factors affecting the properties of supramolecular gel electrolytes was conducted by Dong et al., who used bis(4-acylaminophenyl)methane (structure shown in Figure 6) and bis(4-acylaminophenyl)ether in imidazole-based ionic liquids. It was determined that the length of acyl chains was a strong factor in the resultant gel properties, with longer chains increasing gelation strength but decreasing conductivity [64].

**Figure 8:**
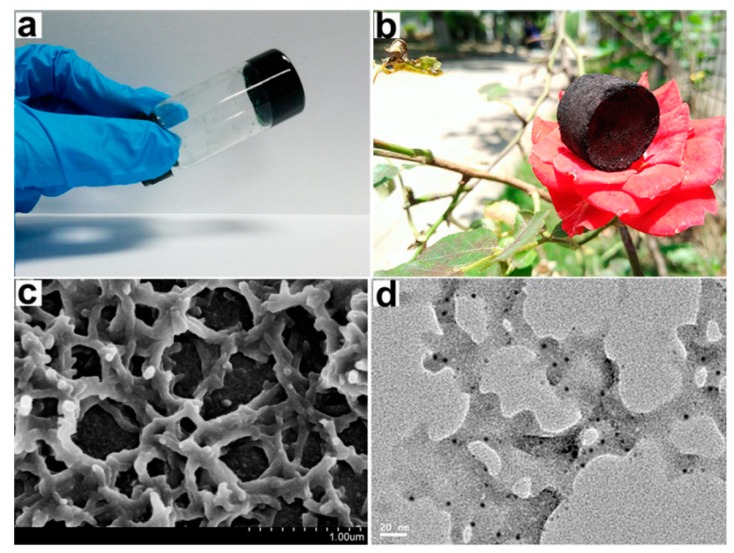
A nanocomposite hydrogel by Liang et al. shown in (**a**) had an extremely low density upon solvent removal, as seen by its aerogel balancing on a flower in (**b**), due to high porosity, as shown in micrographs (**c**) and (**d**). The porosity promotes ion transport and high capacitance [77]. Adapted with permission from ref [77]. Copyright 2015 John Wiley and Sons.

Electrolytes, however, are not the full extent of gel involvement in supercapacitor technology. Supramolecular gels have also found use as supercapacitor electrode materials, as in the work of Liang et al. Their aerogel, comprised of polyaniline with manganese oxide, exhibited qualitative flexibility and 96% retention of capacitance after 20,000 charge/discharge cycles (Figure 8) [77]. Noncovalent interactions, a hallmark of supramolecular gels, were identified to be the source of the gel’s physical strength. Continuing in this vein is the work of Li et al., who worked to create similarly durable and robust conductive hydrogels, and further investigated flexibility. By combining polyaniline and polyvinyl alcohol into a supramolecular hydrogel via dynamic boronic acid group bonding, a capacitance of 306 mF/cm^2^ was achieved in a flexible supercapacitor. While a slightly lower 90% retention of capacitance was shown after 100 charge/discharge cycles, these gels demonstrated complete retention of capacitance after 1000 mechanical folding cycles [78]. Though polyaniline itself is a relatively stiff conductive polymer, its incorporation into a supramolecular gel structure can allow for the tuning of conductive and physical properties to better fit applications. In the realm of graphene-based supercapacitors, similar to the aforementioned idea of a using a supramolecular gel binder coating to prevent the aggregation of anodic nanoparticles in batteries, a gelled structure can improve the quality of devices by preventing π-stacking between graphene layers, which would otherwise decrease ionic diffusion and active surface area [60].

#### 3.2.3. Fuel Cells

Fuel cells present even higher energy density than batteries [76] and greater electrical efficiencies than heat engines [79]. If utilizing water condensation, fuel cells also have the additional benefit of producing no local waste. As such, they are an attractive form of energy storage and have been investigated in the context of supramolecular gels. In the work of Ye et al., supramolecular assemblies of uracil-terminated telechelic sulfonated polyimides (SPI-U) gelled with 20 wt % of an adenine-based hydrogen bonder, dubbed “SMA-A”. The resultant material was used as a proton exchange membrane, allowing for hydrogen ions to pass while excluding methanol. The membranes gelled with SMA-A performed better in terms of stability and selective conductivity than membranes formed of pure SPI, illustrating the benefits of gelation [80]. Outside of electrolytes, Kumar et al. created hydrogels for anode applications in microbial fuel cells. Supramolecular assemblies of graphene oxide (GO), carbon nanotubes (CNT), and poly *N*-isopropylacryl-amide (PNIPAM) hydrogel were created with a range of electronic properties dependent on constituent material ratios. Specifically, it was found that the addition of up to 1% CNT did little to improve the low conductivity of PNIPAM, while the addition of only 0.2% GO increased conductivity by a factor of 10^8^. Interestingly, using the two in concert with 0.1% of each resulted in a 10 times higher conductivity than the previous 0.2% GO sample. This response was attributed to the CNTs inhibiting stacking between GO and increasing contact with the electrolyte. Fuel cells created with the 0.1% GO, 0.1% CNT mixture exhibited a power density around 400 mW/m^2^ over a 300-h operation time [81].

Supramolecular gels have also been of ancillary use to fuel cells in the field of oxygen production. Traditionally, noble metal catalysts (e.g., gold and platinum) have been used for oxygen reduction (ORR) and oxygen evolution reactions (OER), respectively [82], but less costly and more stable materials are desired for more commercially viable fuel cells. In the work of Wu et al., nitric acid, water, and melamine were mixed and heated before the addition of Vulcan XC-72 and cobalt(II) nitrate hexahydrate, which resulted in the gelation of the solution. This supramolecular hydrogel was then dried and heated under a nitrogen atmosphere to yield an assembly of double-shelled Co@CoO@N–C/C nanoparticles. A range of catalysts with differing doping schemes were created using this type of self-assembly process, but Co@CoO@N–C/C was the best alternative catalyst out of their samples due to its close approximation of the performance of traditional platinum/carbon (Pt/C) catalysts while also providing greater stability [83]. A year later, in 2017, Jiang et al. investigated a Co_2_P@CoNPG assembly also synthesized through supramolecular hydrogel methods. This catalyst also exhibited greater stability than Pt/C, retaining 91.6% of its current density after 200 min compared to Pt/C, which presented only 39.3% baseline current density after 200 min in alkaline solution. Intriguingly, this system had the added benefit of effectively catalyzing not just the ORR as the previous example but also the OER [84], opening the door for more expansive utilization of alternative fuel cell catalysts. As research progresses, the ability for supramolecular gels to self-assemble may lead to novel catalytic materials that can outperform and eventually replace noble metals in fuel cell technology.

Here we conclude our discussion on the more physical science-type supramolecular gel applications of optoelectronics and energy devices. We will now delve into the large group of life science applications, starting briefly with the biomedical field mentioned previously.

## 4. Supramolecular Gels for Biomedical Applications

### 4.1. Drug Delivery

The large volume of supramolecular gels investigated for drug delivery applications starkly contrasts that of many other specific applications. Due to their high degree of mechanical, physical, biological, and chemical tunability, supramolecular gels are increasingly found at the cross-section of the next generation of drug delivery materials. Consequently, it should be re-emphasized that this review does not include all examples of supramolecular gel applications, but an overview of these applications with examples of such uses.

#### 4.1.1. Oral Drug Delivery

**Figure 9:**
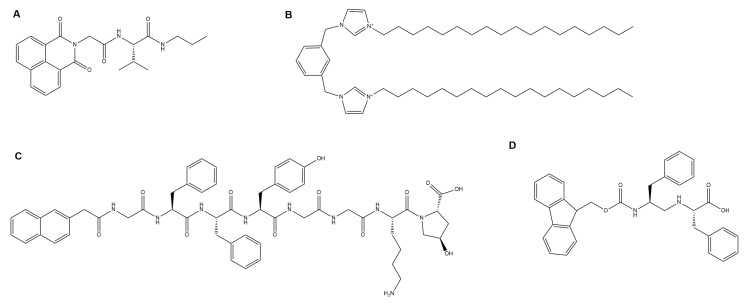
Selected gelators developed for biomedical applications: (**A**) a gelator derived on a 1,8-naphthalimide unit linked to a dipeptidic glycine-valine unit with demonstrated facile transport into human carcinoma cells for oral drug delivery [89]; (**B**) a gemini imidazolium amphiphile gelator for topical drug delivery [90]; (**C**) a collagen-inspired hydrogelator for tissue engineering [91]; and (**D**) an aromatic di-phenylalanine-based gelator silver-based antimicrobial wound treatment [92].

Supramolecular gels have been recognized in oral drug delivery applications for their ability to maintain long-term stability and incorporate environmental sensitivities. Though not explicitly examined in animal models to date, in vitro testing of supramolecular gels promises appropriate properties for ingested drug materials. Hybrid supramolecular gels by Rizzo et al., formed by the self-assembly of Fmoc-l-phenylalanine in the presence of halloysite nanotubes and loaded with the anti-tumor drug camptothecin in aqueous solutions of phosphate buffer saline and phosphate buffer solution, demonstrated notable antiproliferative activity on cervical cancer cells. Camptothecin’s coupling to the supramolecular gel overcame common complications, including the maintenance of the drug in its active form and the prevention of its hydrolysis [85]. Demonstrating the addition of responsiveness for drug delivery applications, a supramolecular gel formed by the host-guest complexation of cucurbit[8]uril and *N*-(4-diethylaminobenzyl)chitosan in 1 M hydrochloric acid by Lin et al. exhibited tunable and pH-sensitive drug delivery rates [86]. Similarly, Ashwanikumar et al. developed a pH-sensitive supramolecular gel with self-assembling RADA-F6 peptides [87] which offered sustained delivery of the anti-tumor drug 5-fluorouracil (5-FU) at basic pH values, making it appropriate for the treatment of colon cancers, among others [88]. Finally, Felip-León et al. produced a supramolecular hydrogel using a low molecular weight gelator based on a 1,8-naphthalimide unit linked to a dipeptidic glycine-valine unit (structure shown in Figure 9) loaded with model dye molecules as particles with dimensions on the nanoscale. These materials showed facile transport into human carcinoma cells, demonstrating the potential for supramolecular gels to be used as an alternative to polymeric nanogels in nanomedicine [89].

With the desirable properties of supramolecular gels for oral drug delivery well established in in vitro studies, further attention should be directed towards elucidating the in vivo behavior of these materials. Considering, e.g., the high salt concentrations which can screen intramolecular interactions and the presence of material-degrading enzymes in animals, future studies should seek to analyze behavior under conditions similar to the desired end use in order to eventually realize clinical applications.

#### 4.1.2. Logic Gate-Based Drug Delivery

**Figure 10:**
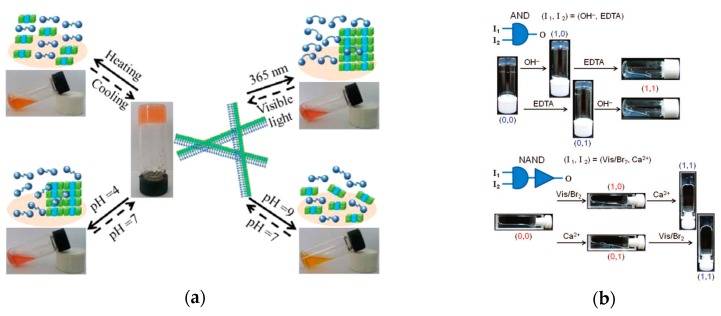
The ability to incorporate several sensitivities into supramolecular gels enables their use in logic-gate based drug delivery. (**a**) OR and XOR functionality in a supramolecular gel by Liu et al. [93]. Adapted with permission from ref [93]. Copyright 2015 American Chemical Society. (**b**) AND, OR, NAND, and NOR functionality in a supramolecular gel by Komatsu et al. [95]. Adapted with permission from ref [95]. Copyright 2009 American Chemical Society.

Incorporating several sensitivities into a supramolecular gel enables its unique use for logic gate-based drug delivery, where quantitative amounts of drugs are released based on input signals. An OR and XOR gate functionality was achieved in co-assembled phenylalanine-based and bis(pyridinyl)-derived hydrogelators by Liu et al. (Figure 10), which use pH, thermal, and light inputs for controlled drug release [93]. Supramolecular peptide-based hydrogel-enzyme hybrids by Ikeda et al. achieved OR and AND functionality to sense specific biochemicals and perform drug release based on the activated logic operation [94]. Accessing several common logic operators, Komatsu et al. realized AND, OR, NAND, and NOR functionality in a supramolecular gel based on a phosphate-type hydrogelator through sensitivities to temperature, pH, the presence of calcium ions, and light (Figure 10) [95].

#### 4.1.3. Topical Drug Delivery

Supramolecular gels have been investigated for several applications in the topical application of anti-inflammatory drugs due to their ease of loading and spreadability, improved drug retention times, and topical cooling effect upon evaporation of the solvent. A bis-imidazolium positively charged amphiphile in ethanol-water mixtures produced by Limón et al. is an example of one such system, with the ability to load and topically deliver anionic drugs, such as non-steroidal anti-inflammatory drugs (NSAIDs). The gel demonstrated rapid drug release profile upon contact with the skin, permitted the permeation of the drug into the skin, and promoted the retention of the drug inside the skin, thus exhibiting properties appropriate for the proposed delivery of anti-inflammatory drugs [96]. Supramolecular gels reported by Klaewklod et al. based on β-cyclodextrin in a low molecular weight polyethylene glycol (400 g/mol) medium with facile spreadability and high flux of the NSAID diclofenac [97], and by Rodrigues et al. using a gemini imidazolium amphiphile (structure shown in Figure 9) in ethanol-water mixtures of several anti-inflammatory anionic drugs [90] fit a similar bill.

Supramolecular gels have been tested for several other topical drug delivery applications. The anti-allergy drug cetirizine was translated into a supramolecular gel in methyl salicylate (menthol) by Majumder et al. with demonstrated efficacy in relieving allergy symptoms in a mouse trial [98]. Marcos et al. formulated a poloxamer-hydroxyethyl cellulose-α-cyclodextrin supramolecular gel in water, which sustained a release of the anti-fungal drug griseofulvin for at least three weeks and demonstrated biocompatibility [99]. To deliver a local anesthetic, prilocaine hydrochloride was loaded into a poly(aryl ether) dendron-based gel in DMSO-water solutions by Kannan et al. for localized, sustained release [100].

Relative to other drug delivery applications, topical drug delivery likely offers the lowest barrier to clinical translation and may offer the entry point for supramolecular gels to reach applied biomedical use. Further development of these materials should include analyses of material toxicity and permeation through skin, and seek to understand the useable lifetime of a topically applied supramolecular gel.

#### 4.1.4. Injectable Drug Delivery

The abilities of supramolecular gels to include temperature-sensitive functionality, rheological tunability, and stability when used as a platform for drug release have garnered interest in their use for injectable drug delivery applications. A thermally-sensitive folic acid-agar supramolecular hybrid gel in a 1:1 DMSO-water mixture by Song et al. demonstrated mechanical tunability and a drug release profile with a low initial burst and long release time with a model drug [101]. With a specific application in mind, Dai et al. developed a supramolecular gel in aqueous media based on host-guest inclusion complexation between α-cyclodextrin and anti-tumor drug-loaded micelles that exhibited several notable properties, including facile preparation, high stability, an attractive drug release profile, low toxicity, and effective in vitro and in vivo anti-tumor activity [102]. Recently, Simões et al. successfully incorporated vancomycin into a Pluronic F127-*co*-α-cyclodextrin supramolecular hydrogel for localized antibiotic treatment of infections. The authors highlighted its long-term stability, ease of syringeability while maintaining viscoelastic properties, and controlled and sustained drug release [103].

### 4.2. Tissue Engineering

Primarily centered around injection-based designs, supramolecular gels have the opportunity to transform tissue engineering by offering minimally invasive materials which can assemble into solid-like structures in the body that are functionalized for relevant high bioactivity. Peptide amphiphiles, which self-assemble into supramolecular gels, are particularly studied due to their inherent bioactivity. Three such examples are highlighted here. Self-assembling peptide amphiphiles in aqueous media by Shah et al. resulted in extensive cartilage regeneration due to surface functionalization with a transforming growth factor β1 binding sequence [104]. Acting as a scaffold for new growth, a guanosine-based hydrogelator by Buerkle et al. offered a self-assembling material with highly tunable mechanical properties with facile injectability and little to no toxicity [105]. Demonstrating the value of supramolecular gel tunability, several collagen-inspired hydrogelators (structure shown in Figure 9) synthesized by Hu et al. controlled peptide sequence and conformation and mechanical properties to optimize cell growth [91].

#### Bone Defect Repair

**Figure 11:**
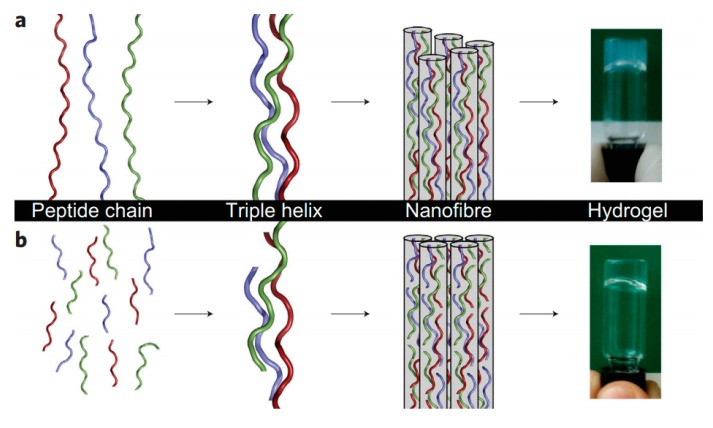
Comparison of self-assembling (**a**) natural rat tail tendon to (**b**) the synthetic collagen-mimetic peptide materials shows notable similarity over several length scales, as demonstrated by O’Leary et al. [108]. Adapted with permission from ref [108]. Copyright 2011 Springer.

As a subset of tissue engineering, supramolecular gels offer similar unique advantages for the repair of critical-sized defects in bone (i.e., defects of a size which cannot be remodeled without intervention). Tested with simvastatin and bone morphogenetic protein, a poloxamine and α-cyclodextrin supramolecular gel in aqueous phosphate buffer by Rosario et al. was successfully able to regenerate bone in rat calvarial defects after injection (and thus notably without surgery) [106]. Self-assembled protein-based microgels by Sang et al. incubated in calcium and phosphate solutions resulted in a hydroxyapatite-mineralized interconnected porous architecture appropriate for bone regeneration [107]. In an effort to closely mimic the properties of bone, O’Leary et al. developed a collagen-mimetic peptide that self-assembled over several length scales into a hydrogel with an organization similar to natural collagen (Figure 11). The properties of the resultant material closely matched that of its natural counterpart, including its breakdown by collagenase, furthering its potential as a bioresorbable replacement material [108].

### 4.3. Silver-Based Antimicrobial Wound Treatment

Facile synthesis routes incorporating silver, a widely accepted antimicrobial agent, combined with unique physicochemical properties have sparked interest in using supramolecular gels for wound treatment. Self-assembling aromatic di-phenylalanine-based materials (structure shown in Figure 9) loaded with silver by Paladini et al. were developed for use as a wound dressing by maintaining a moist aqueous environment that promotes wound healing while preventing infections with low silver concentrations [109]. A silver-infused l-lysine-based amphiphilic hydrogelator by Mandal et al. combined mechanical rigidity with a shear-thinning ability for application as an injectable hydrogel. Notably, the hydrogel was toxic towards bacteria without interrupting the growth of mammalian cells [110]. Similarly, an l-cysteine-based hydrogel by Pakhomov et al. demonstrated toxicity at low silver concentrations across numerous bacteria cultures, all while maintaining appropriate mechanical properties for wound and burn treatment [92].

### 4.4. Non-Viral Vector Delivery

A fundamental challenge underlying genetic therapies is the lack of safe yet effective vectors for delivering foreign DNA into cells. While viral vectors are highly effective for this purpose, concerns over their safety have instigated great caution in their use. Conversely, while synthetic counterparts are preferred for their lower immunogenicity and higher loading ability, their significantly lower efficiency has prevented successful clinical translation [111]. Supramolecular gels offer the opportunity to overcome these challenges through facile formulations of highly tunable materials that remain in the application site (i.e., gel in vivo) for sustained transduction. As this area of research is in its infancy, only one recently reported system is currently published, though it is expected that many more will arrive in the literature as these novel characteristics are exploited. The existing example of this application belongs to Rey-Rico et al., who developed a polypseudorotaxane supramolecular gel in phosphate-buffered saline with demonstrated ability to transfect recombinant adeno-associated virus (rAAV) vectors into human mesenchymal stem cells while maintaining suitable mechanical robustness over time periods of relevance. The addition of α-cyclodextrin into the gels resulted in elevated rAAV concentrations and maintained transgene expression [112]. Further expansion of the field into this application can unlock new pathways for gene delivery, a historically difficult but important challenge.

## 5. Supramolecular Gels for Biological Applications

### 5.1. Cell Storage and Study

Fibrous supramolecular gels offer tunable biochemical, mechanical, and physical properties with dimensions on the order of the extracellular matrix (ECM), making them ideal candidates for use in cell storage and study. Self-assembling peptide-based hydrogels are perhaps the most common derivative within this class, offering biomimetic and bioactive building blocks for assembly. With a controllable number of exposed arginylglycylaspartic acid (RGD) tripeptide sequences on the surface, a hydrogel scaffold developed by Zhou et al. is one such classic example of this application, offering a stiff and highly hydrated ECM-like scaffold for anchorage-dependent cells [113].

**Figure 12:**
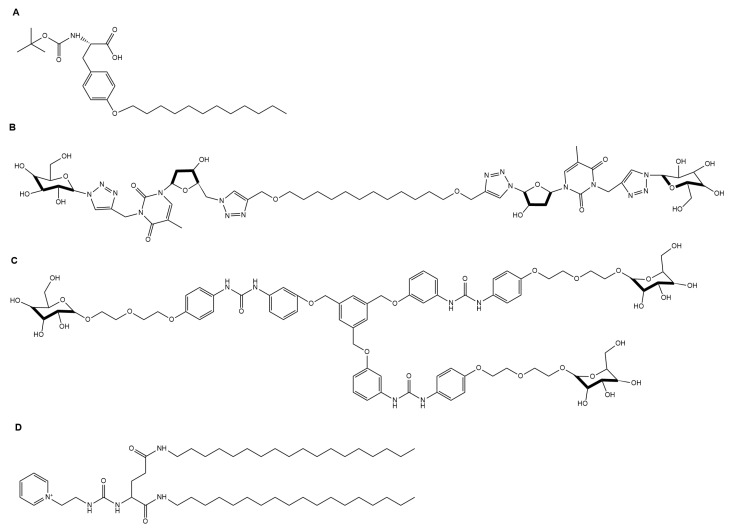
Selected gelators developed for biological applications: (**A**) a Boc-*O*-dodecyl-l-tyrosine gelator for the cryopreservation of cells [115]; (**B**) a bole-amphiphile gelator for cell culturing [116]; (**C**) a tris-urea hydrogelator for electrophoresis [117]; and (**D**) a cationic pyridinium-glutamide amphiphilic gelator for selective visual indication of the presence of adenosine triphosphate (ATP) [118].

**Figure 13:**
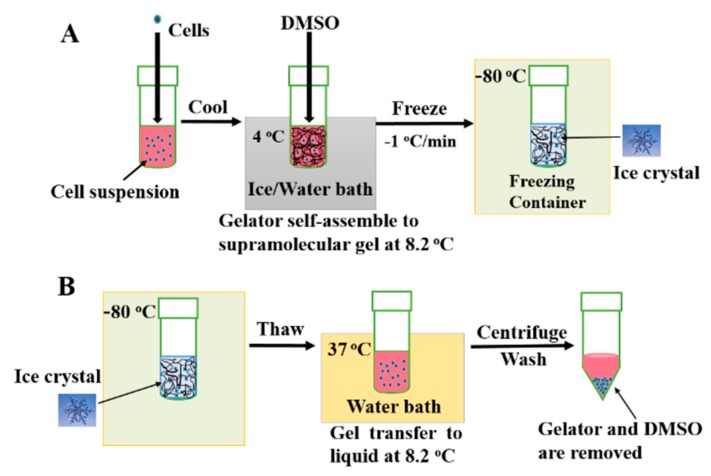
The (**a**) cooling and (**b**) warming processes of the supramolecular gel developed for minimizing cell damage during storage via cryopreservation by Zeng et al. [115]. Adapted with permission from ref [115]. Copyright 2015 John Wiley and Sons.

Though the cryopreservation of cells is established as an effective long-term cell storage method for cells, in which cells are brought to subzero temperatures to arrest and thawed to regain biological activity, the process offers several opportunities for damaging cells. These include the formation of intracellular and extracellular ice crystals, cytotoxicity from cryoprotective agents, and toxicity from highly concentrated electrolytes and solutes due to ice formation [114]. A novel Boc-*O*-dodecyl-l-tyrosine (BDT) supramolecular gel system (structure shown in Figure 12) in a culture medium developed by Zeng et al. overcame many of these issues by confining ice crystal growth into the gel pores, lowering cell volume change rates, depressing osmotic shock, and decreasing the volume of crystalline ice produced (Figure 13). Furthermore, the thermoreversibility of the system (unique to supramolecular gels) allows for the facile removal of the gel, as the material is in its liquid state at room and biological temperatures [115].

Material stiffness is well recognized as a key influencer in many dynamic biological processes, such as cell differentiation, tissue development, and tumor progression [119]. A supramolecular hydrogel developed by Zheng et al. allowed clinical studies of such dynamic processes through light modulated stiffness [120]. This hydrogel, which stiffens upon the application of near infrared light and expands upon the application of ultraviolet light, offers spatiotemporal resolution of a critical matrix parameter.

A low molecular weight hydrogelator system developed by Poolman et al. demonstrated the versatility of supramolecular gel systems. Through a self-described “toolbox” of modifications to a base hydrozone gelator, their hydrogel could be functionalized with proteins for increased bioactivity or modified for multicolor imaging of mammalian cells and their substructures nested in the gel network [121].

#### Stem Cell Study and Differentiation

Because of the highly tunable nature of supramolecular gels, much interest has been garnered in developing these gels to direct and study stem cell differentiation. A low molecular weight bole-amphiphile gelator (structure shown in Figure 12) published by Latxague et al. self-assembled in water into cell culture scaffolds, with transmission electron micrograph and rheological results indicating that the 3D matrix shape affects the behavior of stem cells [116]. Jacob and Ghosh et al. furthered this work with the development of an amyloid nanofibril hydrogel which supported cell attachment and spreading over a range of cell types for cell culturing and offered tunable stiffness that notably directed differentiation of mesenchymal stem cells [122]. Lastly, Alakpa et al. showed perivascular stem cells cultured on supramolecular peptide gels of 1, 13, and 32 kPa stiffnesses differentiated into neuronal, chondrogenic, and osteogenic cells, respectively. Then, analyzing deviations in the concentration of over 600 metabolites during differentiation on the latter two scaffolds, the authors discovered that a specific lipid was significantly depleted in each process, demonstrating the utility of supramolecular gels for identifying cell-directing metabolites [123].

The tunability of supramolecular gels offers the unique ability of cell testing in a manner similar to that acheived by Alakpa et al. Through thoughtful design of the constituents included in the gel structure, supramolecular gels may be used for detailed and insightful analysis of cellular processes ranging from proliferation to endocytosis to movement.

### 5.2. Electrophoresis

Gel electrophoresis is extensively used in biological fields to separate macromolecules such as DNA, RNA, and proteins based on their size and charge [124]. Today, sodium dodecyl sulfate polyacrylamide gel electrophoresis (SDS-PAGE) enjoys widespread use for this characterization method. However, supramolecular gels offer notable enticing advantages over their polyacrylamide gel counterparts; namely, significantly easier separation of proteins from the matrix and the ability to tune separation based on the structure of the matrix [125]. Dubbed SDS-SUGE (SDS-supramolecular gel electrophoresis), the low molecular weight tris-urea hydrogelator (structure shown in Figure 12) matrix developed by Yamamichi et al. provides a prime example for the separation of proteins and their facile recovery through centrifugation [117]. A study by Munenobu et al. further demonstrated that the separation of acidic proteins on the same matrix was based on isoelectric point rather than molecular weight and that the proteins retained their native forms during and activity after separation [126]. Highlighting the efficiency of this system relative to SDS-PAGE, Tazawa et al. demonstrated that SDS-SUGE could be used in a continuous field manner for the separation of DNA fragments up to 166 kbp, whereas a pulsed field, requiring specialized equipment and a long analysis time, typically must be used for a conventional polyacrylamide matrix [127]. The notable advantages of SDS-SUGE over its conventional counterpart may disrupt a well-established bioanalytical technique in favor of tunability and protein recoverability.

### 5.3. Biological Compound Indicator

The collapse of supramolecular gel structures in the presence of specific biological compounds offers enticing applications for the facile detection of these complex molecules without instrumentation. The fabrication of a supramolecular gel from a cationic gelator (a pyridinium tethered covalently to a glutamide amphiphile) (structure shown in Figure 12) and the visual indicator methyl orange in a 1:1 mixture by mole of ethanol and water by Yang et al. selectively collapsed in the presence of the key cellular energy molecule adenosine triphosphate (ATP) but not the other related adenosine-phosphates adenosine diphosphate (ADP) and adenosine monophosphate (AMP), allowing visual discriminations between these key metabolic molecules [118]. Similarly, a cholesterol-based gel in dimethylformamide (DMF)-water mixtures prepared by He et al. for amino acid detection selectively collapsed in the presence of aspartic acid and glutamic acid [128]. A precursor which self-assembled to a hydrogel upon enzyme catalysis was reported by Yang and Xu. When the enzyme was mixed with possible inhibitors before contacting the precursor, actual inhibitors prevented gel formation, and their corresponding minimum inhibition concentration could be determined in a way amenable to running a parallel assay to simultaneously test many inhibitors [129]. Supramolecular gels may thus be developed into rapid, useful tests for commonly performed biological assays.

## 6. Special Topic Applications of Supramolecular Gels

Though they do not explicitly fit into the other broad categories previously discussed in this review article, supramolecular gels are found at the forefront of research for several additional specific industrial applications. These special topics are discussed here and demonstrate the versatility of supramolecular gel materials to span the breadth of scientific fields.

### 6.1. Environmental Remediation

#### 6.1.1. Water Treatment

**Figure 14:**
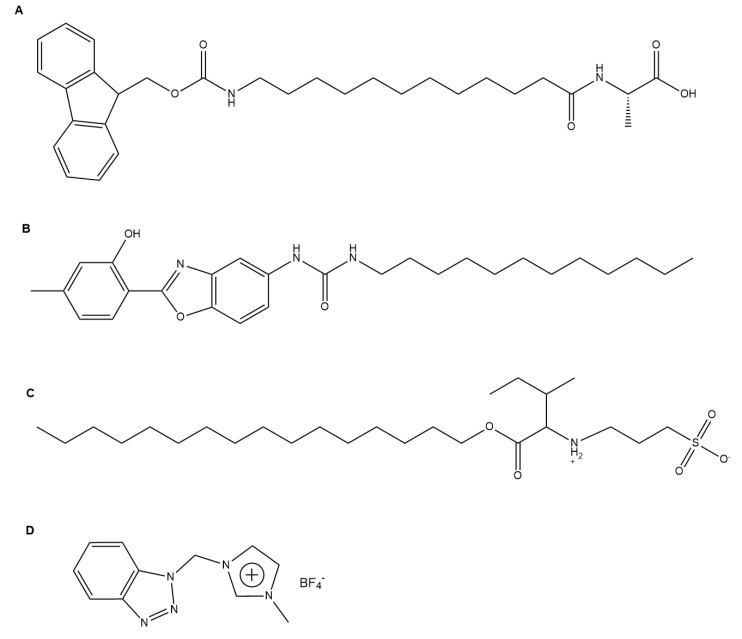
Selected gelators developed for special topic applications: (**A**) an ambidextrous bifunctional hydrogelator for the removal of toxic cationic and anionic organic dyes from water [130]; (**B**) an organogelator containing 2-(2′-hydroxyphenyl)benzoxazole for the visual detection of nerve agents [133]; (**C**) a zwitterionic gelator for tribology [134]; and (**D**) an imidazolium-based gelator containing a benzotriazole group for anticorrosion and lubrication [135].

The functionalization, high aspect ratio, and high loading capacities of supramolecular gels make them ideal candidates for developing the next generation of water treatment materials. For example, an ambidextrous bifunctional hydrogelator (structure shown in Figure 14) synthesized by Basu et al. showed the ability to absorb toxic cationic and anionic organic dyes from contaminated water, and demonstrated continued viability over several cycles [130]. Considering the widespread use and presence of mercury despite its toxicity, Lin et al. developed a naphthalimide-functionalized supramolecular polymer gel whose corresponding xerogel showed a 98% removal rate of Hg^2+^ and recyclability [131]. A comprehensive purification system was proposed by Yan et al., in which a two component tetrazoyl derivative-octadecylamine supramolecular gel assembled in cyclohexane rapidly co-adsorbed cationic dyes, anionic dyes, and heavy metal ions with demonstrated recyclability and reusability and without significant decreases in efficiency [132].

Additional functionalities have been investigated for developing a supramolecular gel which not only cleans water, but can easily be added to and removed from the solution. Zeng et al. incorporated Fe_3_O_4_ nanoparticles into a benzyl sorbitol-derived supramolecular gel reinforced with halloysite nanotubes and assembled in a 4:1 by weight ethanol-water mixture with dye absorptive properties. In addition to increasing the strength of the gel, the nanoparticles allow for the facile removal of the gels from the solutions they purify by applying a magnetic field [136]. A ferrocene-containing supramolecular gel assembled in heptane by Yan et al. efficiently removed iodine from water and was able to undergo a shear-induced gel-sol transition, offering a new method for water purification. Shaking the gel into its sol phase allows it to disperse throughout the matrix, and, within minutes, the gel is reformed containing high quantities of extracted iodine. The gel can then be directly removed, leaving behind purified water [137].

The scalability and maintenance of the gelled structure under mechanical stress are of the highest consideration as the field moves towards developing and testing further supramolecular gels for water remediation. Whether a supramolecular gel can withstand significant shear flow, what the maximum rate of aqueous flow through the gel is, and to what degree these gels can be made cost effective will guide their implementation into applied use.

#### 6.1.2. Oil Recovery

**Figure 15:**
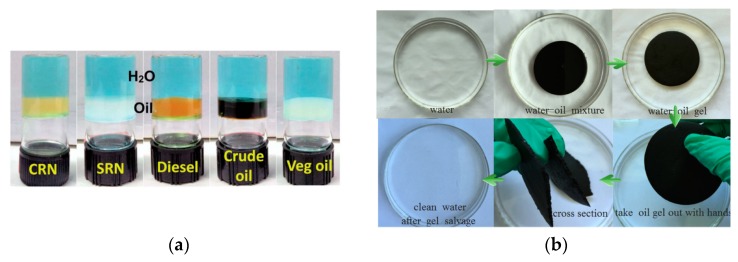
Supramolecular gels have been designed for the highly selective separation of crude oil products from water. (**a**) The separation of several oils from water with a xylitol-based organogelator by Raju et al. [141] Adapted with permission from ref [141]. Copyright Year Copyright Owner’s Name. (**b**) The segregation and removal of Russian crude oil from water using an alkyl bicarbamate supramolecular gel by Wang et al. [140]. Adapted with permission from ref [140]. Copyright 2017 Elsevier.

The annual transportation of millions of tons of petroleum across waterways worldwide puts the ecosystems they cross at dire risk, particularly as spills are unpredictable and the resources to mitigate their impact are often not readily available [138]. Supramolecular gels offer a unique opportunity to combat this issue through the selective gelation of oil products on orders of magnitude larger than their own weight. A dipeptide-based gelator by Raju et al. highlighted this application well, with the phase-selective gelation solely of crude oils with varying gravities and refinery products in mixtures with water. Notably, this system was able to absorb up to 166 times its own weight in hydrocarbons [139]. Similarly, an alkyl bicarbamate supramolecular gel by Wang et al. (Figure 15) showed rapid (within 15 min) selective removal and the retention of oils from oil-water mixtures [140]. For a complete cycle of recovery, a xylitol-based organogelator by Raju et al. (Figure 15) demonstrated not only the phase-selective gelation of oil products, but the recovery of oil from the gels as well [141].

As with water treatment, scalability looks to be the most pressing concern for these gelator materials. Though the efficacy of these materials is remarkable for oil recovery, vast quantities would be required for real-world oil spill remediation. Supramolecular gels for this application should be further tested in turbulent conditions, simulating the open ocean, to determine when gelation readily occurs. Additionally, the toxicity of these materials should be determined, as quantities which do not participate in the gelation of oil would likely remain in the environment.

#### 6.1.3. Chemical Warfare Agent Sensing and Decontamination

Perhaps one of the most sobering developments of supramolecular gels is their tuning for the detection and collection of chemical warfare agents. Kim et al. noted this application in 2010 with the synthesis of an organogel containing 2-(2′-hydroxyphenyl)benzoxazole (structure shown in Figure 14) in chloroform, which enables the detection of nerve agents through demonstrated sensing of the organophosphate diethyl chlorophosphate (DCP) through a visual colorless to yellow color shift of the gel [133]. Hiscock et al. developed a system sensitive to dimethyl methylphosphonate (DMMP) and highly sensitive to soman through the interruption of the sol-gel transition in their tren-based tris-urea supramolecular gel system assembled in toluene [142]. It should be noted that while DCP and DMMP are not themselves nerve agents, they are commonly used in laboratories as a safe analog to agents such as sarin, soman, and tabun because of their parallel reactivity [143].

Hiscock et al. went on to develop oximate-containing organogels in dimethyl sulfoxide, which could decontaminate organophosphorus liquid agents and sense the presence of liquid and vapor agents through color changes. The collapse of the gels in the presence of large volumes of warfare agents could subsequently be employed to release localized bursts of a decontaminant [144]. The incorporation of metal oxides into supramolecular gels in the presence of peroxides, notably using off-the-shelf reagents, can alternatively be used for the catalytic degradation of warfare agents, such as the reported degradation of the gaseous sulfur mustard stimulant 2-chloroethyl ethyl sulfide [145].

### 6.2. Participation in Synthesis

#### 6.2.1. Catalysis

The high surface area-to-volume ratio of nanostructures, which in turn imparts high reactivity, and the controllable fabrication of supramolecular gels have made sparked interest in their use as scaffolds for catalytic applications, improving upon existing industrial synthesis procedures. Self-assembling glycyrrhizic acid gelators incorporated with graphene oxide and gold nanoparticles in water by Saha et al. illustrated the catalysis of several electron transfer processes, such as the reduction of *p*-nitrophenol to *p*-aminophenol [146]. l-glutamic acid-based hydrogelators developed by Jin et al. offered a similar promise when complexed with Cu^2+^ for the catalysis of Diels-Alder cycloaddition reactions, with an accelerated reaction rate and improved control of product chirality relative to existing Cu^2+^-containing nanostructures [147]. Finally, a xerogel formed by the complexation of Cd^2+^ with a cyclohexane-based gelator by Lee et al. exhibited activity as a base catalyst for the Knoevenagel condensation reaction [148]. Though all gels presented here utilized a cofactor for catalysis, it should be noted that peptide-based self-assembling gelators are being increasingly studied for catalytic applications due to their inherent specific reactivity [149].

#### 6.2.2. Template for Crystal Growth

Gels have historically been used for templating crystal growth because they impart improved optical properties, larger sizes, and fewer defects than crystals grown in the absence of a gel [150]. Supramolecular gels improve upon conventional gel templates because they offer readily reversible and stimuli-tunable sol-gel transitions and can gel in a wider variety of solvents. Foster et al. realized four such bis(urea) gelators which offered controlled growth of a diverse range of pharmaceuticals in a variety of organic solvents, including carbamazepine, acetaminophen, and ibuprofen. In many cases, an anion-triggered gel dissolution allowed for the facile recovery of the drug [151]. Further work by Foster et al. demonstrated that a bis(urea) supramolecular gel in toluene could be used for the polymorph control of grown pharmaceutical crystals [152]. In an unrelated line of research, Daly et al. demonstrated the growth of single-crystal halide nanowires on a supramolecular gel scaffold through a diffusion-driven base growth mechanism; the growth of such structures could not be facilitated by conventional gel scaffolds [153].

### 6.3. Surface Coatings

#### 6.3.1. Tribology

Friction and wear frequently stress and damage mechanical equipment while causing unnecessary energy consumption. Lubricants are commonly employed to address these concerns; however, traditional lubricating oils suffer from leakage, volatilization, and climb shift. Semi-solid greases created as an alternative to oils face oil separation and poor lubrication from the use of a thickener [154]. Consequently, supramolecular gels have been investigated as an intermediate material between oils and greases to overcome the systemic problems of each category. A zwitterionic gelator (structure shown in Figure 14) in base oil by Yu et al. is one such example of this application, which achieved evident friction reduction while maintaining stable mechanical properties and rapid creep recovery [134]. Similarly, a thiol-functionalized polyhydric gelator in base oil by Huang et al. offered superior lubrication properties at steel-steel, steel-copper, steel-aluminum, and steel-alumina interfaces [155]. Further supramolecular gels by Yu et al. based on amino acid-derivative gelators in base oils showed superior tribological properties to common greases and oils while forming a protective film that prevented the direct contact and consequent severe wear of sliding pairs [156].

#### 6.3.2. Anticorrosion

Ionic liquid gels formed through supramolecular self-assembly offer intriguing use for anticorrosion applications, often while maintaining favorable tribological properties. Cai et al. synthesized an imidazolium-based gelator containing a benzotriazole group (structure shown in Figure 14), which in turn endowed anticorrosion and antioxidant properties to the material. The thixotropic properties of the gel further offered potential use of the material in high performance lubrication applications, such as for electrical contacts [135]. A similar imidazolium-based gelator with a urea group by Yu et al. offered desirable anticorrosion and tribological properties with facile attachment to metal surfaces resulting from its urea-based and imidazole ring functional groups [157].

As many tribologically-minded supramolecular gels use similar structures and functionalities to achieve friction resistance to those seen for anticorrosion applications, it seems relevant that many of the former gels be tested for the latter properties. Reporting the additional anticorrosive functionality of these gels can accelerate their pace towards commercial acceptance.

### 6.4. Fat Replacement in Consumer Food Products

Societal, governmental, and industrial pressures to reduce or eliminate saturated and trans fats from consumer food products have sparked interest in using supramolecular gels to create structured liquid oils as an alternative fat source to hard fats or liquid oils structured with saturated fatty acids [158]. Edible self-assembling small molecule gels are specifically of interest for this application, where small molecules act as the gelator and oils act as the solvent. Co and Marangoni developed one such system with 12-hydroxystearic acid to gel vegetable oils, with shear and thermal fields used to tune the material’s mechanical properties and consequent mouth-feel [159]. Similarly, Alvarez-Mitre et al. formulated self-assembling 12-hydroxystearic acid-based ionic gelators for the gelation of safflower oil [160]. Short chain ceramides analyzed by Rogers et al. not only demonstrated the gelation of edible oils like canola oil, but surprisingly decreased the viability of colon, prostate, ovarian, and leukemia cancer cell lines due to the bioactivity of the gelator, demonstrating the translational ability of their material [161]. Such reports highlight the potential of supramolecular gels to disrupt well-established fat sources in the name of consumer health.

## 7. Conclusions

Supramolecular gels have found use in a wide breadth of applications over the past two decades due to their tunable mechanical, electrical, biocompatible, and chemical properties and self-healing abilities. While supramolecular gels have exhibited many specific breakthroughs, further improvements in increased device lifetimes, lower cost and higher throughput processing, and knowledge of long-term biological and environmental effects are needed before these applications can become staple products of a new generation of materials. Additionally, other areas such as logic gate applications, biomimetic prostheses, and non-viral vector gene delivery are still in early development stages. Further research in the field promises the use of supramolecular gels as functional soft materials, forging avenues toward widespread flexible electronics, smarter sensors, revolutions in alternative energy, novel medical treatment methods, greater biological understanding, and expanded industrial capabilities.
